# Application of Intelligent Inspection Robot in Coal Mine Industrial Heritage Landscape: Taking Wangshiwa Coal Mine as an Example

**DOI:** 10.3389/fnbot.2022.865146

**Published:** 2022-06-23

**Authors:** Yan Shen, Yu Li, Zengping Li

**Affiliations:** ^1^Art School, Northwest University, Xi'an, China; ^2^School of Automation Science and Electrical Engineering, Beihang University, Beijing, China; ^3^Xi'an Edson Landscape Design Co., Ltd, Xi'an, China

**Keywords:** industrial heritage protection, intelligent inspection robot, underground planning, artificial intelligence, mine

## Abstract

This study is developed to explore the role of intelligent inspection robot in the protection and utilization of coal mine industrial heritage. Based on the actual situation of the coal mine, the underground planning protection scope is analyzed. Aiming at the problems of imperfect fire early warning detection technology, management mechanism, high labor cost and low work efficiency in underground protection, the intelligent inspection robot technology is proposed to realize safety tour, underground intelligent management and early warning of underground security, fire protection facilities construction, and intelligent early warning system. This paper analyzes the key technology of intelligent inspection robot in coal mine industrial heritage protection, introduces the composition, structure and implementation method, and proposes its construction path and method. Besides, the path planning, motion obstacle avoidance and sensing detection of the robot are studied. The research shows that the intelligent inspection robot has comprehensive functions and stable performance, and can realize the scientific, intelligent and refined management of industrial heritage protection, which provides a guiding basis for the intelligent protection of coal mine industrial heritage.

## Introduction

Under the background of global resource depletion and urbanization, the problem of coal mine wasteland has become 1 of the factors restricting the sustainable development of society, economy and ecology (Li et al., 2015; Hu and Lin, 2016; Chang et al., 2017; Xiao et al., 2018; Marko et al., 2020). On the 1 hand, with the disappearance and transfer of old industries, some industrial structures, plant buildings, and their supporting facilities began to lose their original significance. Using the progress of science and technology to eliminate the negative environmental effects of coal mine wasteland and realize safe and efficient protection and utilization is the development direction of coal mine industrial heritage, and the traditional protection mode needs to be changed (Gregorová et al., 2020; Konior and Pokojska, 2020). On the other hand, with the rapid development of science and technology and the popularization of artificial intelligence technology, this is conducive to optimizing the coal mine industrial structure, increasing industrial safety. We put into use “few people or unmanned” intelligent robots, so as to improve scientificity and efficiency (Wen et al., 2013; Ge, 2014; Malte et al., 2019). It can be seen that the application of artificial intelligence technology is also an inevitable choice for the regeneration of environmental landscape in coal mine wasteland.

At present, the theoretical research on coal mine intelligent inspection robot mainly focuses on coal mine security control and intelligent mining. It is mainly summarized into 2 aspects: 1 is to emphasize the use of industrial internet to reduce the labor intensity of inspection workers and improve the inspection quality (Zhu et al., 2013). Besides, aiming at the inspection requirements of dangerous gases such as gas in special areas under the coal mine, we try to realize unmanned detection of dangerous areas by optimizing robot inspection technology (Li et al., 2020). Ge et al. (2020) proposed the coal mine robot system and key technologies. Lu et al. (2015) designed a state inspection robot to realize state inspection (Zhang and Yue, 2015; Sun, 2016). Hao and Yuan (2020) designed automatic inspection robot,which can replace manual inspection on fully mechanized mining face. Existing studies have broadened the cognitive scope of coal mining security, so that there is no dead angle and zero blind area in mine safety monitoring (Wang et al., 2018; Zhu et al., 2019). Applying it to the protection and utilization of coal mine wasteland is not only conducive to the promotion of intelligent mine, but also can reduce the impact of human error and strengthen safety management. However, there is still a lack of systematic theoretical research and extensive practical exploration on how to take the coal mine wasteland as a landscape resource and use the intelligent inspection robot technology (Zhang et al., 2020) and 5G technology (Jun et al., 2021) to further excavate its cultural landscape value, aesthetic value and economic driving value on the basis of underground heritage security and detection.

Therefore, through the theoretical and practical research on intelligent inspection robot, we focus on the path planning, motion obstacle avoidance and sensing detection of coal mine inspection robot, and discuss the security line and sensing detection method of underground intelligent inspection robot in coal mine wasteland. Through research and practice, it is found that coal mine intelligent inspection robot can reduce labor cost and improve work efficiency; ensure the safe and stable operation. The research results have important application value for improving the security ability and digital management of coal mine industrial heritage and promoting the sustainable development of coal mine wasteland.

## Engineering Background

### Regional Characteristics

**Figure 1:**
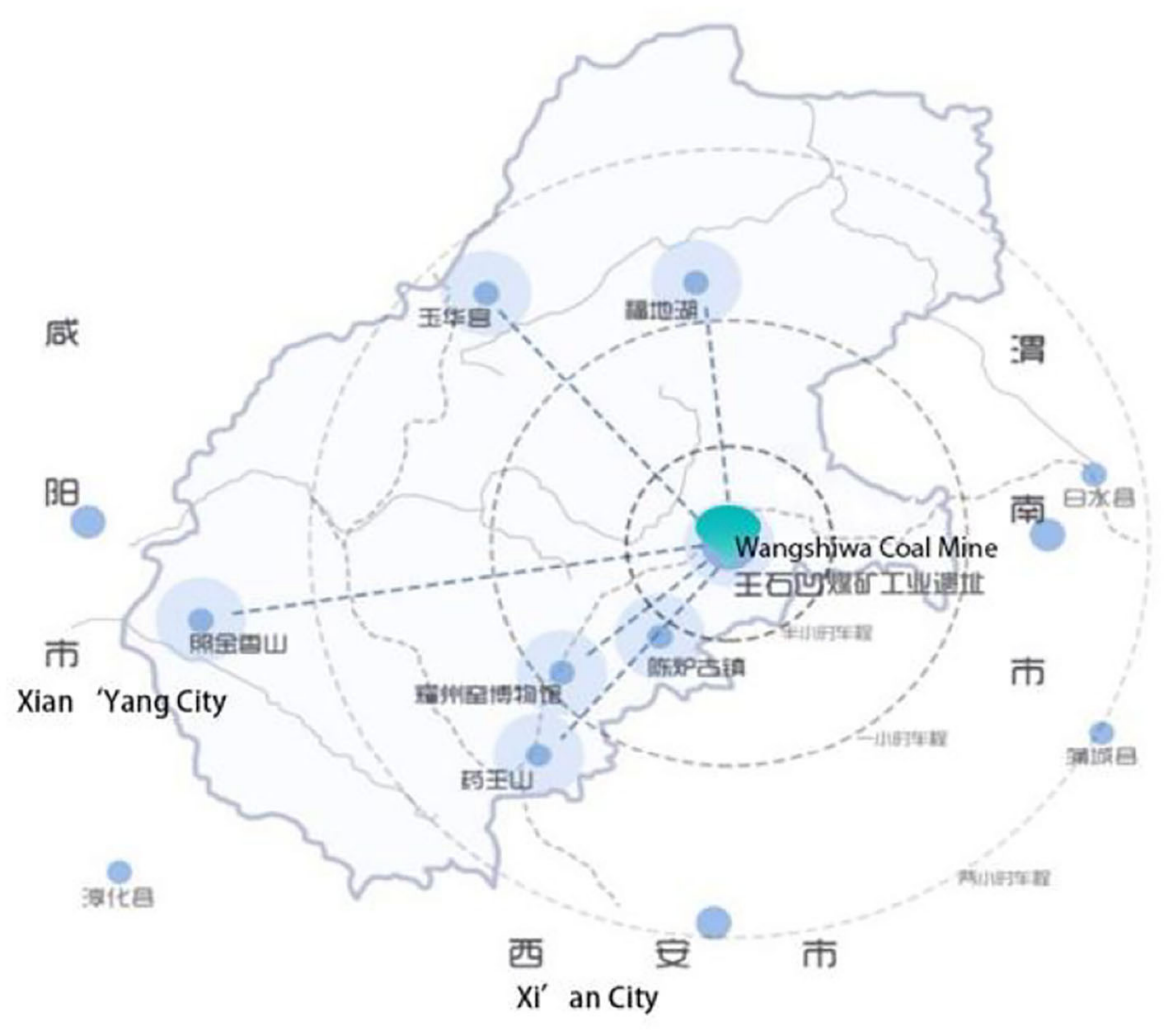
Location map of Wangshiwa Coal Mine.

**Figure 2:**
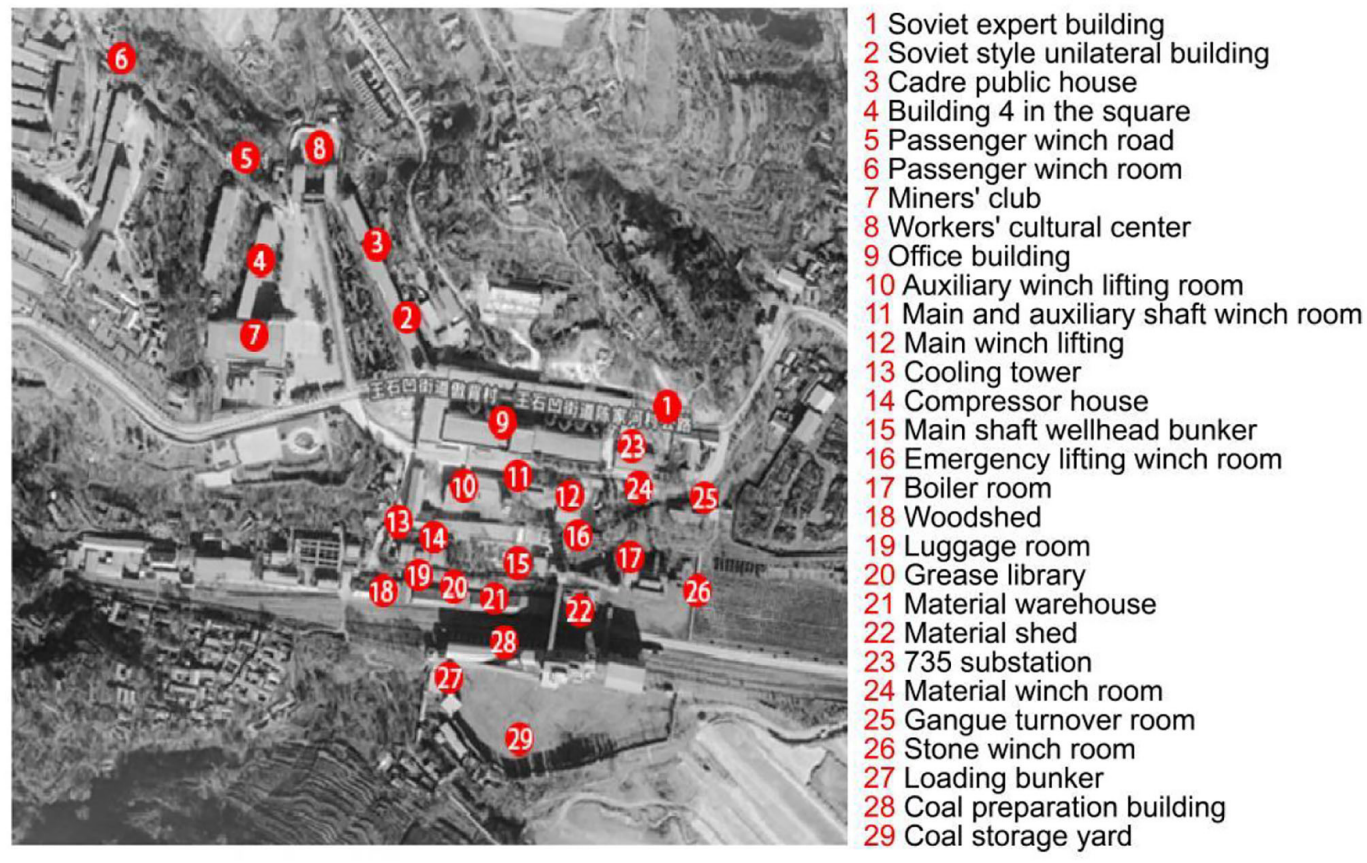
Layout of Wangshiwa Heritage.

**Figure 3:**
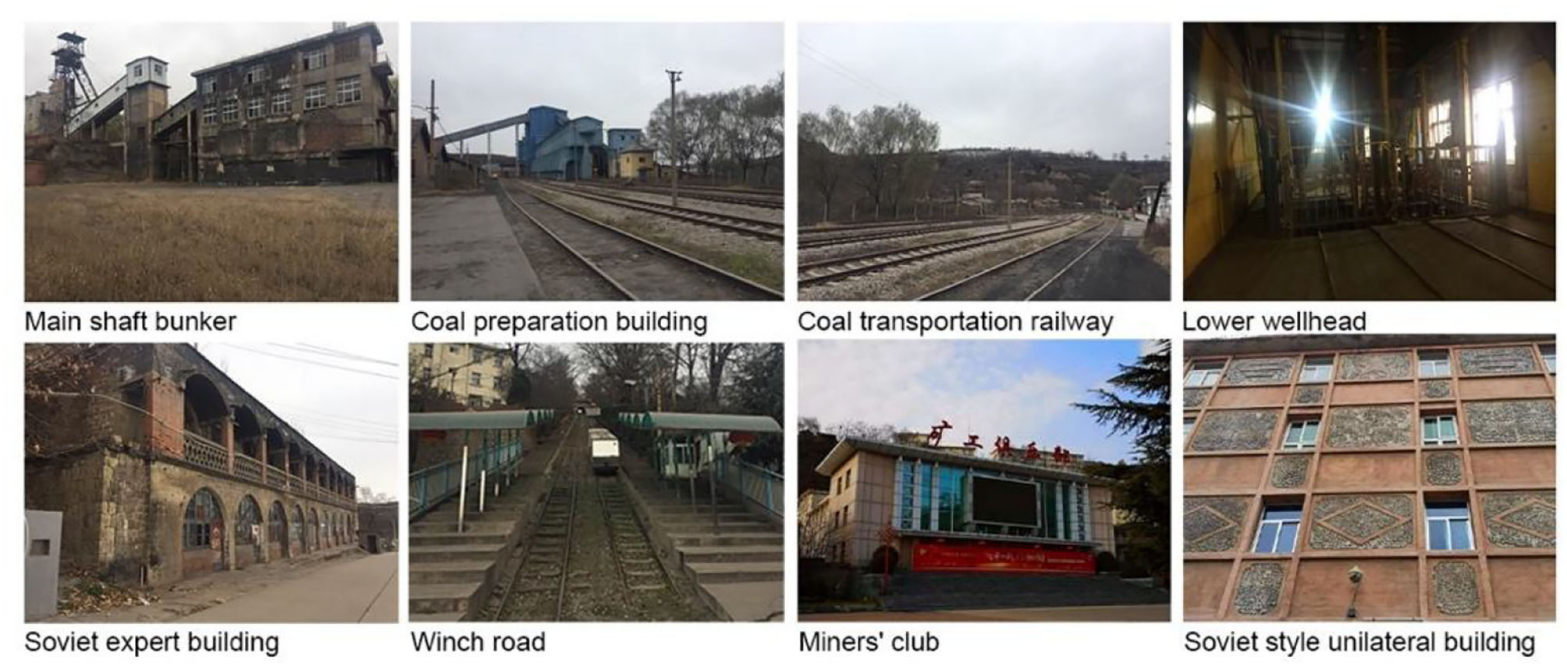
Current situation of Wangshiwa Coal Mine.

The Wangshiwa industrial heritage is taken as the research object, as shown in Figures 1–3. Wangshiwa Coal Mine is located 12.5 kilometers away from the eastern suburb of Tongchuan City, Shaanxi Province, China (Records of Tongchuan mining bureau (1990-2015), 2015). It is 1 of the 156 key engineering construction projects in the first 5-Year Plan of new China (Wang, 2014). With resource exhaustion, weakened market demand and other factors, the coal mine cannot continue to be exploited and was officially closed in 2014 (Tongchuan city annals compilation committee Tongchuan city annals., 1997).

### Underground Planning and Protection Scope

At present, the underground part reserved 735 underground car yard areas while other areas at 735 levels have been closed and 650 levels has been closed. Considering that the closed underground area was permanently closed, the closed area was not taken as the protection object in the plan. The details of Wangshiwa coal mine heritage are shown in Tables 1, 2.

**Table 1:** Production facilities list of Wangshiwa Coal Mine.

**Facilities**	**Facility name**	**Construction age**	**Facility function**	**Preservation** **situation**	**Service** **condition**	**Protection measures**
Auxiliary shaft	Auxiliary shaft	1959	Manual lifting	Better	Used	Appearance repair
	Auxiliary shaft	1959		Common	Used	
	Auxiliary shaft wellhead	1961		Better	Used	
	735 roadway	1959	Underground vegetable shed	Worse	Used	Structural stability assessment, maintenance, protection and repair
	Main shaft hoisting machine room	1959	Raw coal lifting	Better	Used	Appearance repair, protection and repair
	Main shaft	1959		Common	Used	
	Main shaft wellhead bunker	1959		Better	Used	
	Raw coal belt	1961	Raw coal screening	Common	Used	Building mapping, structural stability assessment, appearance repair
	Coal preparation building	1961		Common	Used	
	Coal return belt	1961		Common	Used	
	Lump coal belt	unknown		Common	Used	
	Supporting buildings of coal preparation building	1961		Common	Used	
Production auxiliary facilities	Compressor house	1958	Compressed air system	Better	Used	Building mapping, structural stability assessment, and appearance repair
	735 central water tank	1959	Drainage system	Better	Used	Structural stability assessment, and building mapping
	735 substation	1959	Power supply system	Common	Used	Structural stability assessment, and building mapping

**Table 2:** Overview of Wangshiwa underground heritage.

**Function**	**Name**	**Date of initial construction and transformation**	**Protection measures**	**Exhibition and utilization planning**
Underground mining	735 roadway	1959	Structural stability assessment, protection and repair	Coal mining technology viewing room, underground cinema, etc.

### Problems in the Protection of Underground Cultural Relics

(1) The security and fire protection system is imperfect. First, the original security and fire protection facilities in the mining area are set up to meet the needs of the production period. So they cannot meet the protection requirements of cultural and museum units. Secondly, the existing mine patrol inspection mostly relies on the inspection personnel to carry flashlight and detection equipment to regularly walk in the complex roadway for inspection. The gas concentration at that time and whether there are roof fall, roof leakage and other special conditions in the roadway are noted on the fixed-point board, reported manually.

(2) Manual inspection incurs human errors and staff safety is not guaranteed. Some working faces use fixed-point cameras to assist patrol inspection, and transmit the video or picture information collected by the cameras to the upper computer. The staff can understand the underground security situation through real-time monitoring. For the inspection of harmful combustible gas, we can arrange combustible gas or harmful gas detection devices in the coal mine underground and configure alarm system (Xu et al., 2010). These require the staff to have a high degree of professionalism, and the underground inspection personnel have work risks.

(3) Traditional manual inspection is still adopted. Traditional inspection methods include manual inspection and online monitoring. Manual inspection has high-labor intensity and low efficiency. Although the online monitoring method has good detection effect, it has high cost, poor flexibility and small coverage area (Zuo, 2012; Lu et al., 2015; Ge and Zhu, 2017; Pei et al., 2017).

At present, in view of the shortage of manual inspection and potential risks, it is urgent to adopt intelligent means to protect and manage the underground landscape and human safety. Intelligent inspection robot system is introduced to reduce the influence of human error, strengthen production safety management, and improve work efficiency.

## Key Technologies of Coal Mine Inspection Robot

**Figure 4:**
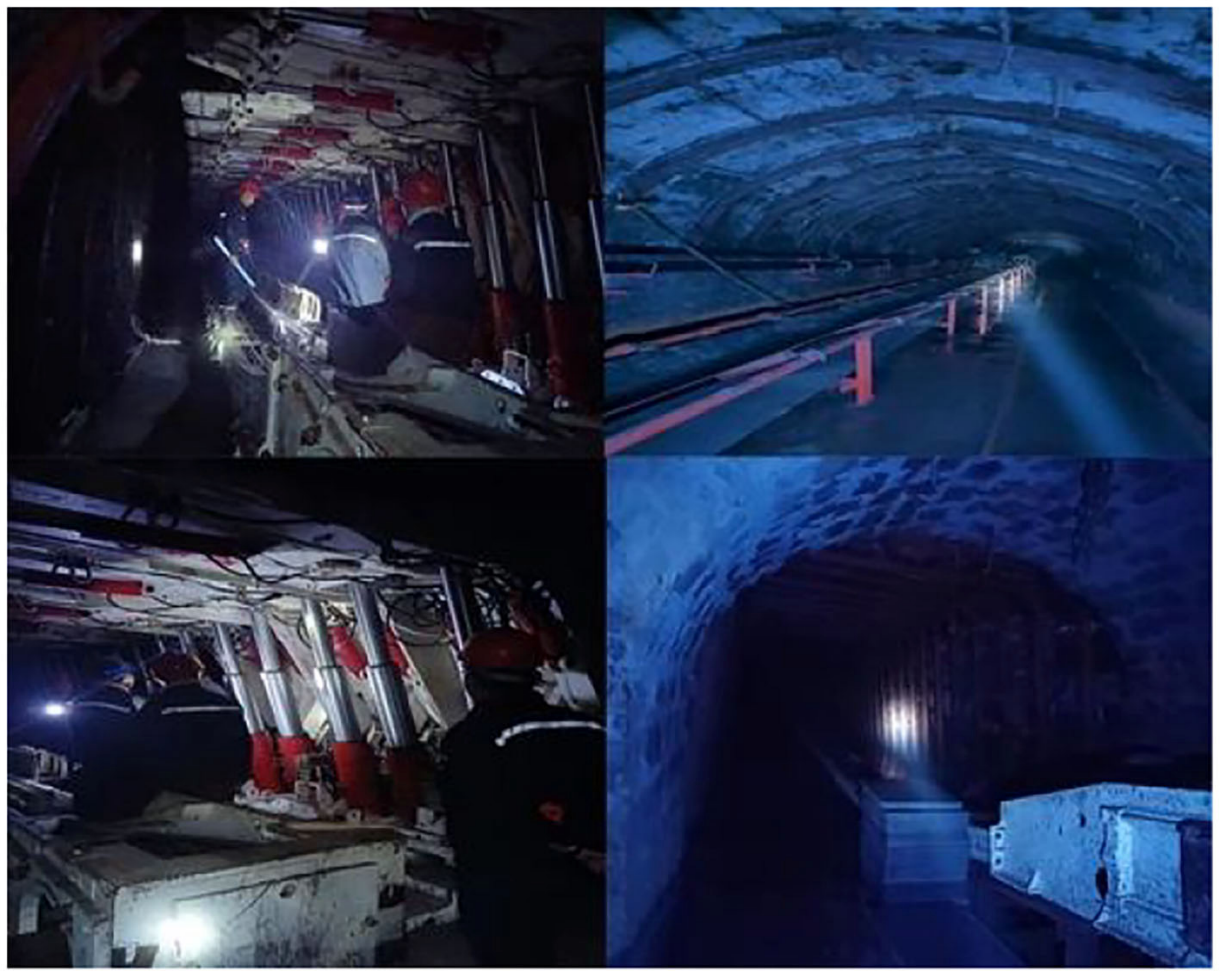
Current situation of underground coal mine.

**Figure 5:**
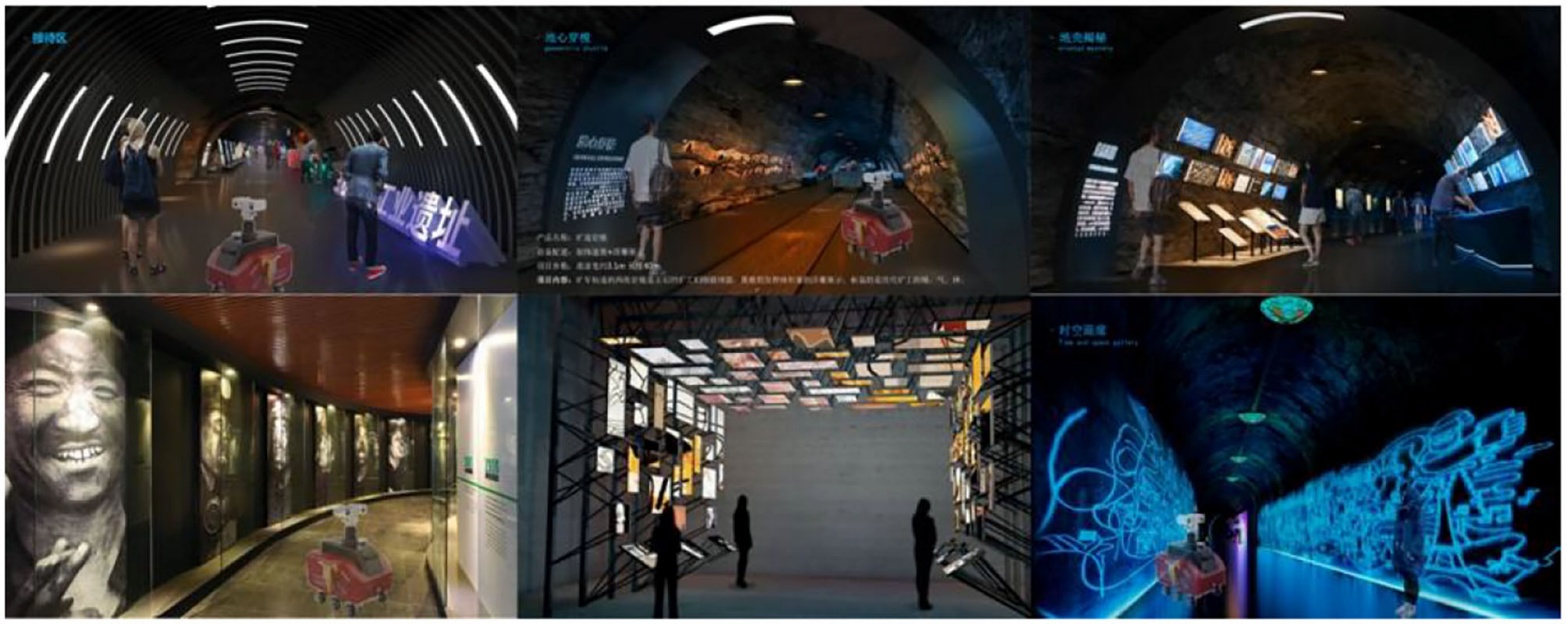
Underground landscape regeneration design of coal mine.

Field exploration was carried out in the underground Wangshiwa Coal Mine, as shown in Figure 4. Spatial landscape design was carried out according to the actual site environment, as shown in Figure 5.

According to the underground reconstruction, the inspection robot is applied to the industrial heritage after the transformation of the mine, mainly for the detection of lines and electronic equipment. For this purpose, robot path planning, motion obstacle avoidance, and sensing detection methods are proposed.

### System Composition and Technical Route of Inspection Robot

**Figure 6:**
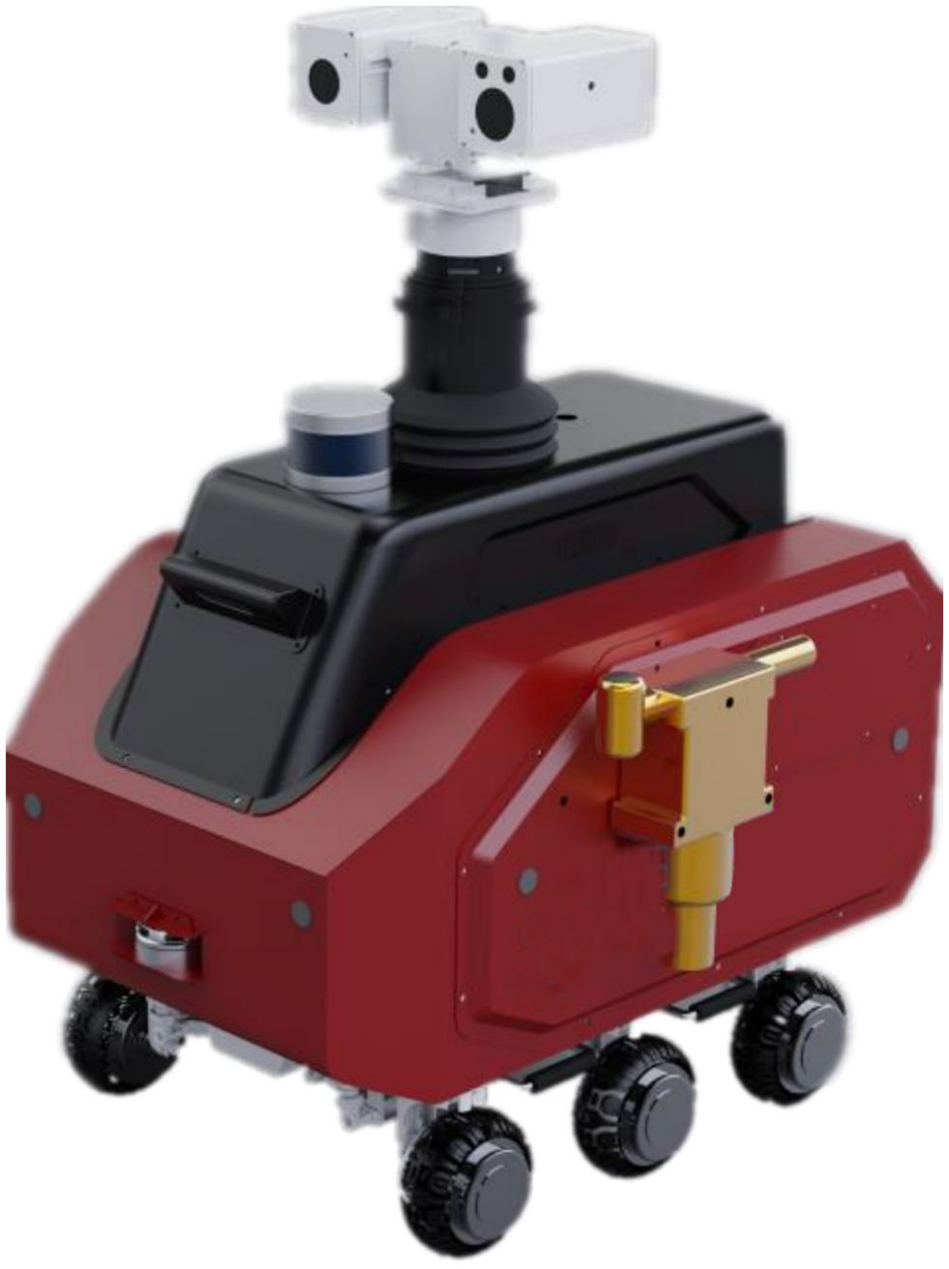
The 6-wheeled robot.

The inspection robot is mainly composed of 5 parts: robot hardware system, robot software system, operation and maintenance management cloud platform, PC client, and Android client. The 6-wheeled robot is shown in Figure 6.

**Figure 7:**
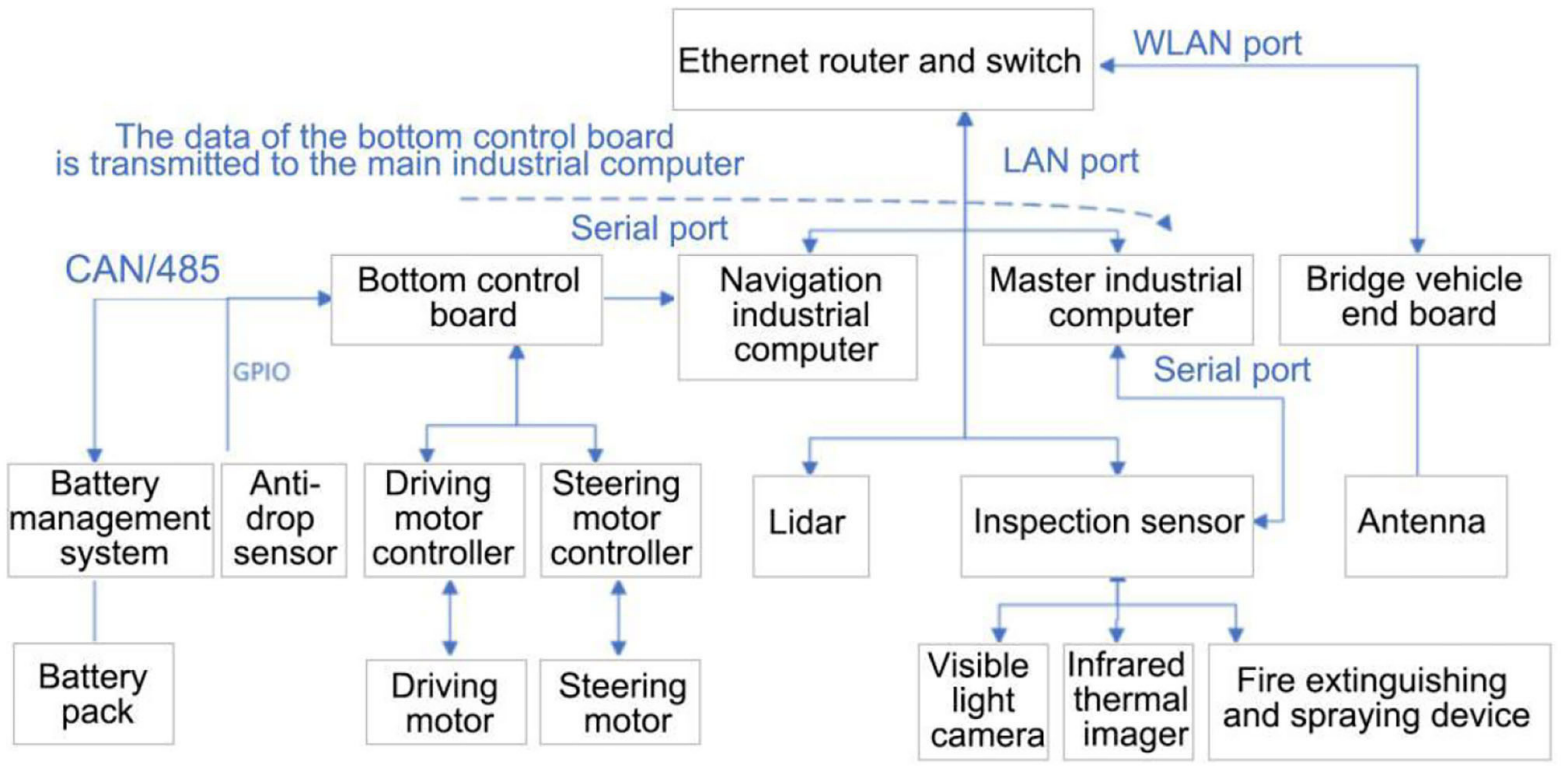
Composition block diagram of vehicle body.

The inspection robot adopts 4-wheel chassis, and is equipped with visible light camera, infrared thermal imager, laser radar, audible and visual alarm, and other equipment. The internal connection relationship is shown in the Figure 7.

**Figure 8:**
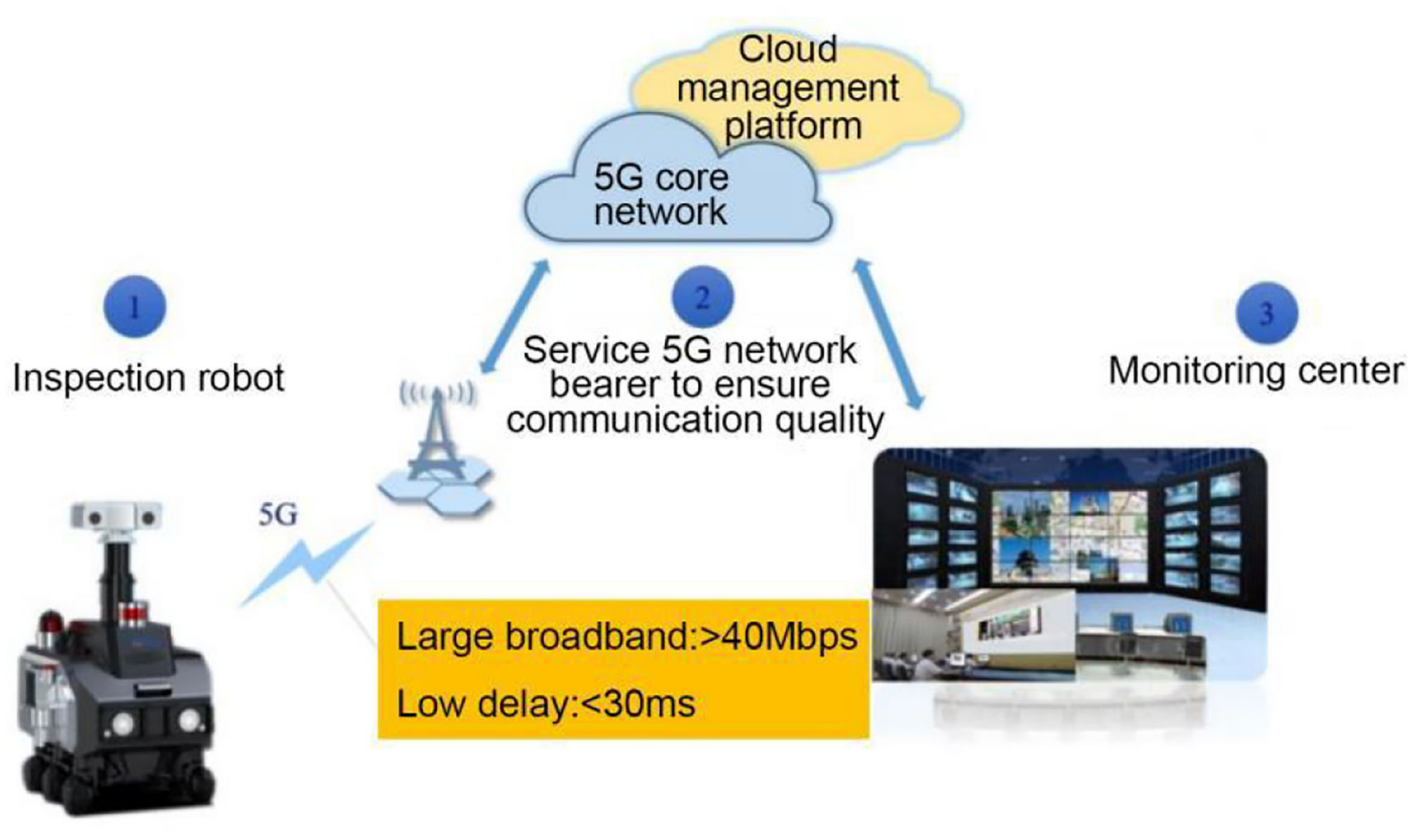
Composition of inspection robot system.

The inspection robot is mainly composed of the robot body (hardware system and software system) for data collection and processing, and uploads the information to the operation and maintenance management cloud platform through 5G/4G network (Zhen et al., 2021). The information is displayed to the operation and maintenance personnel through the WEB client and Android client. The operation and maintenance personnel can also send tasks to the robot through the client. If there are abnormal phenomena, such as over-temperature, the robot sends the alarm information to the management cloud platform for operation and maintenance personnel to view and deal with, as shown in Figure 8.

### Path Planning

According to the underground reconstruction planning, path planning and motion obstacle avoidance of inspection robot is an important research content.

#### Path Planning

A^*^ algorithm is proposed for the global path planning problem of static map. According to the lidar scanning, the map data is obtained, the grid map environment model is established, and then the A^*^ algorithm is used for preliminary path planning. A^*^ algorithm is a typical heuristic search algorithm. The method is simple and fast, and the heuristic search is much targeted. It only needs to search part of the state space. The algorithm has small search range, low search complexity and high path search efficiency. In A^*^ algorithm, the direction of guided search and decision through the evaluation function is expressed as:


$$\begin{array}{l} f\left(n\right)=g\left(n\right)+h\left(n\right) \end{array}$$

*f*(*n*) represents the valuation function from the starting point to the target point. *g*(*n*) is the movement cost from the starting point to node *n*. *h*(*n*) is the estimated cost from the node to the target node *n*. The value of each function can be obtained through the above function. The optimal node is selected by selecting the node with the lowest value, which directly determines the search of the optimal path of the algorithm.

When the algorithm is implemented, it is represented by grid map based on inspection task. The map environment includes starting position, passable area, non passable area, target area, etc.

A^*^ algorithm has good real-time performance when conducting path planning experiments in static environment, so it can quickly and efficiently solve the optimal path. In the design of A^*^ algorithm, this paper uses the Euclidean distance as the heuristic function.


$$\begin{array}{l} h\left(n\right)=\sqrt{{\left(x_{i}-x_{n}\right)}^{2}+{\left(y_{i}-y_{n}\right)}^{2}} \end{array}$$

The abscissa and ordinate of the starting point in the grid map are *x*_*i*_ and respectively. The abscissa and ordinate of the end point in the grid map are *x*_*n*_ and *y*_*n*_ respectively.

#### Obstacle Avoidance

In the inspection process, robots should avoid obstacles effectively to avoid collisions. The obstacle avoidance task is realized by using artificial potential field method. By introducing the concept of “mechanical field,” the robot's operating environment is constructed as an environment with artificial potential field, that is, artificial gravitational field is constructed at the planned target point, and artificial repulsive field is constructed around the obstacle. In the artificial potential field, the robot's path planning and search for the collision free path is to search for the descending direction of the potential function.

**Figure 9:**
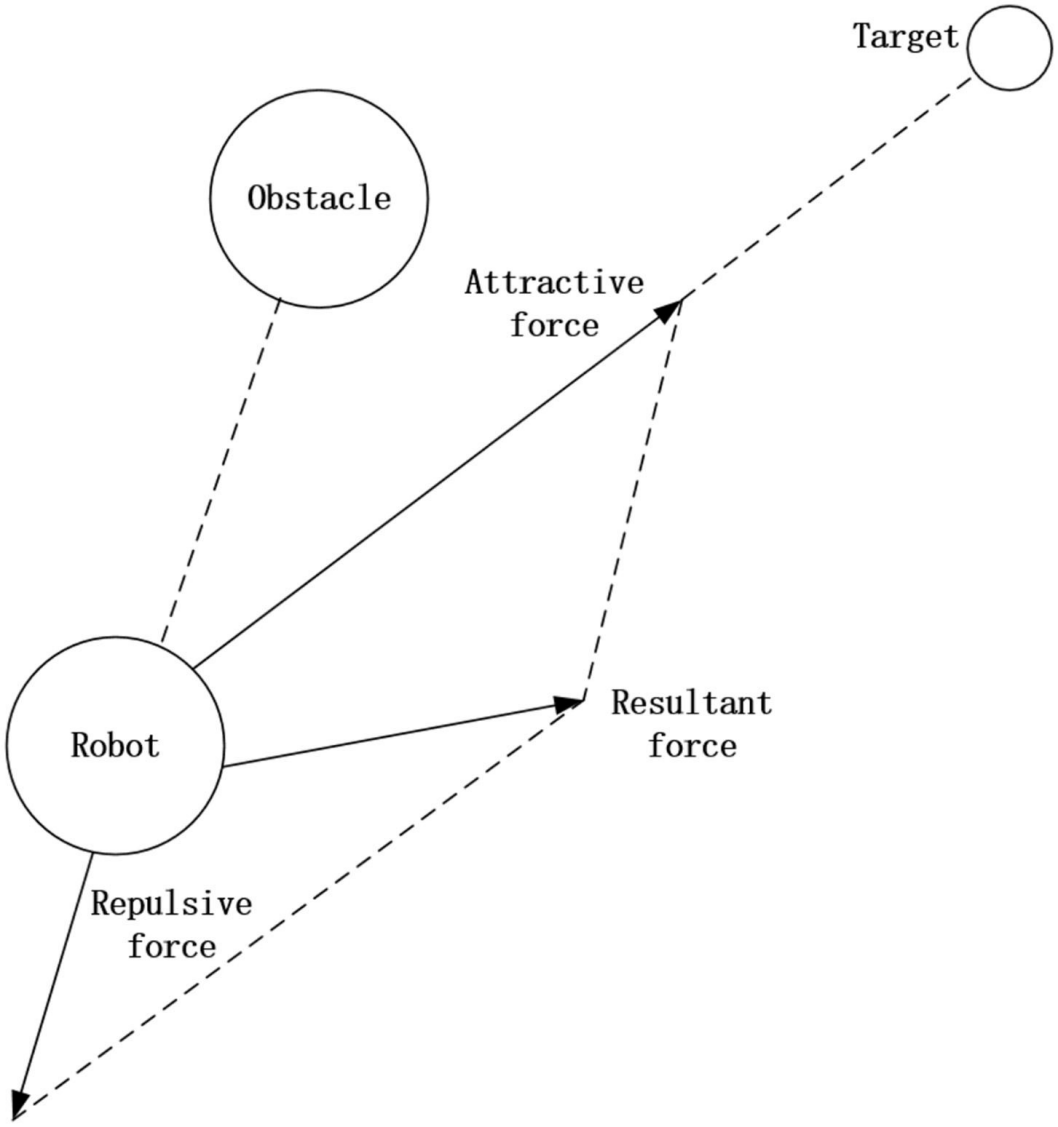
Force analysis diagram of robot in potential field.

When the robot is in the artificial potential field, the force analysis diagram of the gravity from the target point and the repulsion from the obstacle is shown in Figure 9.

Based on the artificial potential field method, the virtual repulsive force *F*_*rep*_ acting on the connecting link is introduced. The minimum distance is *d*_min_, the maximum distance is. When the distance satisfies *d*_min_<*d*<*d*_safe_, the virtual repulsive force increases with the decrease of distance, which can be set as


$$\begin{array}{l} F_{rep}=\eta \left(\frac{1}{d}-\frac{1}{d_{safe}}\right) \end{array}$$

where η is the repulsive scale factor. So the expression of virtual repulsive force is


$$\begin{array}{l} F_{rep}=\{\begin{array}{c} F_{\max}\\ \eta \left(\frac{1}{d}-\frac{1}{d_{safe}}\right)\\ 0 \end{array}\begin{array}{c} ,\\ ,\\ , \end{array}\;\begin{array}{c} d<d_{\min}\\ d_{\min}<d<d_{safe}\\ d>d_{safe} \end{array} \end{array}$$

**Figure 10:**
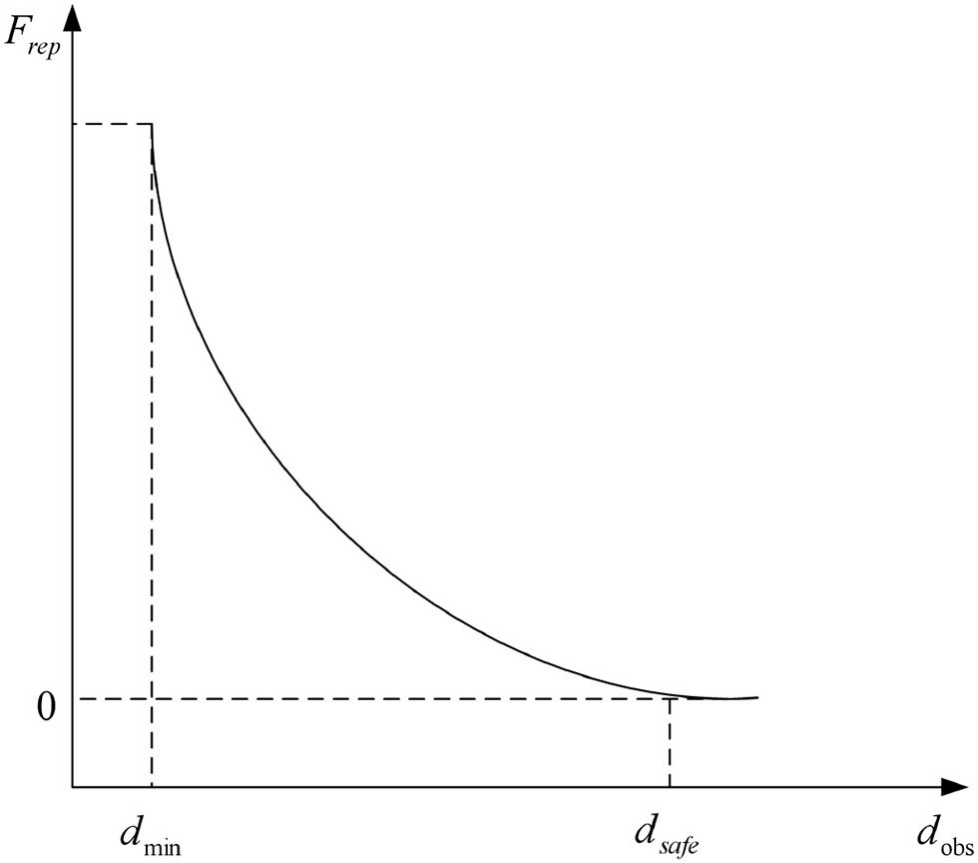
Variation curve of virtual repulsive force with distance.

The variation curve of virtual repulsive force with obstacle distance is shown in Figure 10.

In the potential field environment where the robot is located, the vector sum of the gravitational force of the target point and the repulsive force of the obstacle determines the motion direction of the robot. The vector sum can make the robot move toward the predetermined target point and avoid the collision. Obstacle avoidance is realized by detecting distance.

#### Sensing Detection

The relevant sensors on the robot are used to perform detection tasks and judge whether there are anomalies, including the following aspects.

(1) Temperature measurement function. Measure the temperature of the wiring terminal and the distribution cabinet of the battery pack. The cabinet body to be measured is identified by visible light camera. Then the temperature is judged and recorded by correction and analysis of infrared thermal image and visible light image. The temperature difference between the 2 times will be compared in the next inspection, and the alarm will be generated when the temperature difference exceeds alarm warning temperature.

(2) Circuit breaker status identification. Identify the circuit breaker status based on the deep learning algorithm through the visible camera, and report the status to the monitoring platform after identification.

Circuit breaker state recognition belongs to image recognition, and convolutional neural network method can be used. The circuit breaker image is preprocessed to obtain the contour image of the circuit breaker.

The features of the preprocessed circuit breaker image are then calculated to obtain the image's feature vector. The circuit breaker feature vector is then fed into a convolutional neural network to generate an abstract feature vector. The feature vector of the actual circuit breaker image is input to realize the recognition of the circuit breaker state.

The recognition model of deep learning algorithm based on convolutional neural network is not sensitive to external interference, so the recognition accuracy is high and the matching accuracy can reach more than 90%.

(3) Meter identification. It can identify voltmeters, ammeters and other meters, and upload the meter display to the monitoring platform. If the meter display is abnormal, it can give an alarm in time.

(4) Status indicator recognition. Judge the number and position of status indicators through the visible camera based on the deep learning algorithm, and judge the current status of indicators in combination with the thermal infrared sensor.

(5) Environmental monitoring. The ambient temperature, humidity and gas are detected by carrying an environmental sensor, and an alarm is given if the preset value is exceeded.

Based on the inspection requirements of mine industrial heritage lines and electronic equipment, the highly intelligent robot technology, and image recognition technology are used to complete the inspection and diagnosis of equipment running status in special environment instead of manual work.

### Underground Layout and Inspection Path

**Figure 11:**
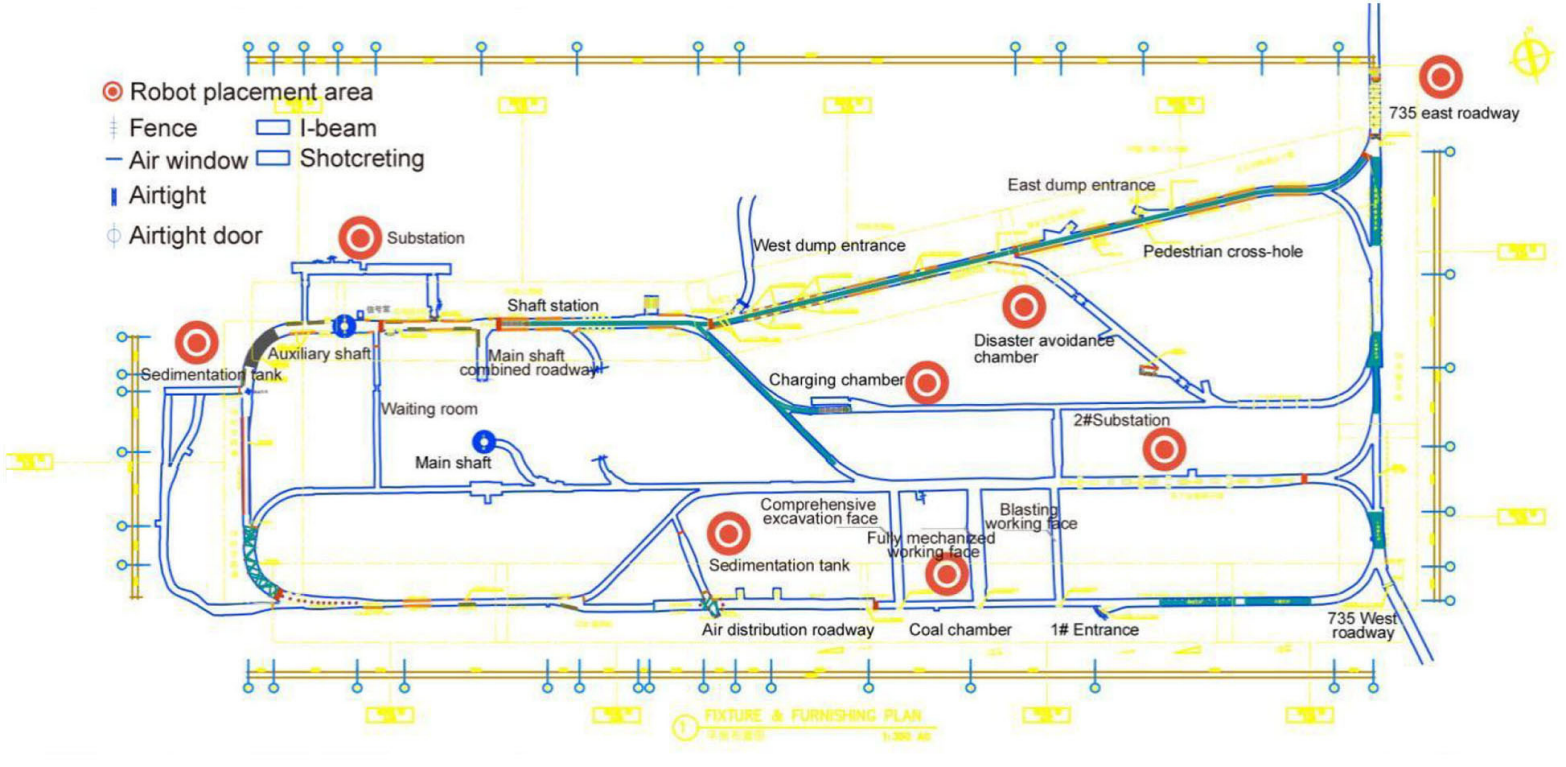
The diagram of inspection system distribution.

**Figure 12:**
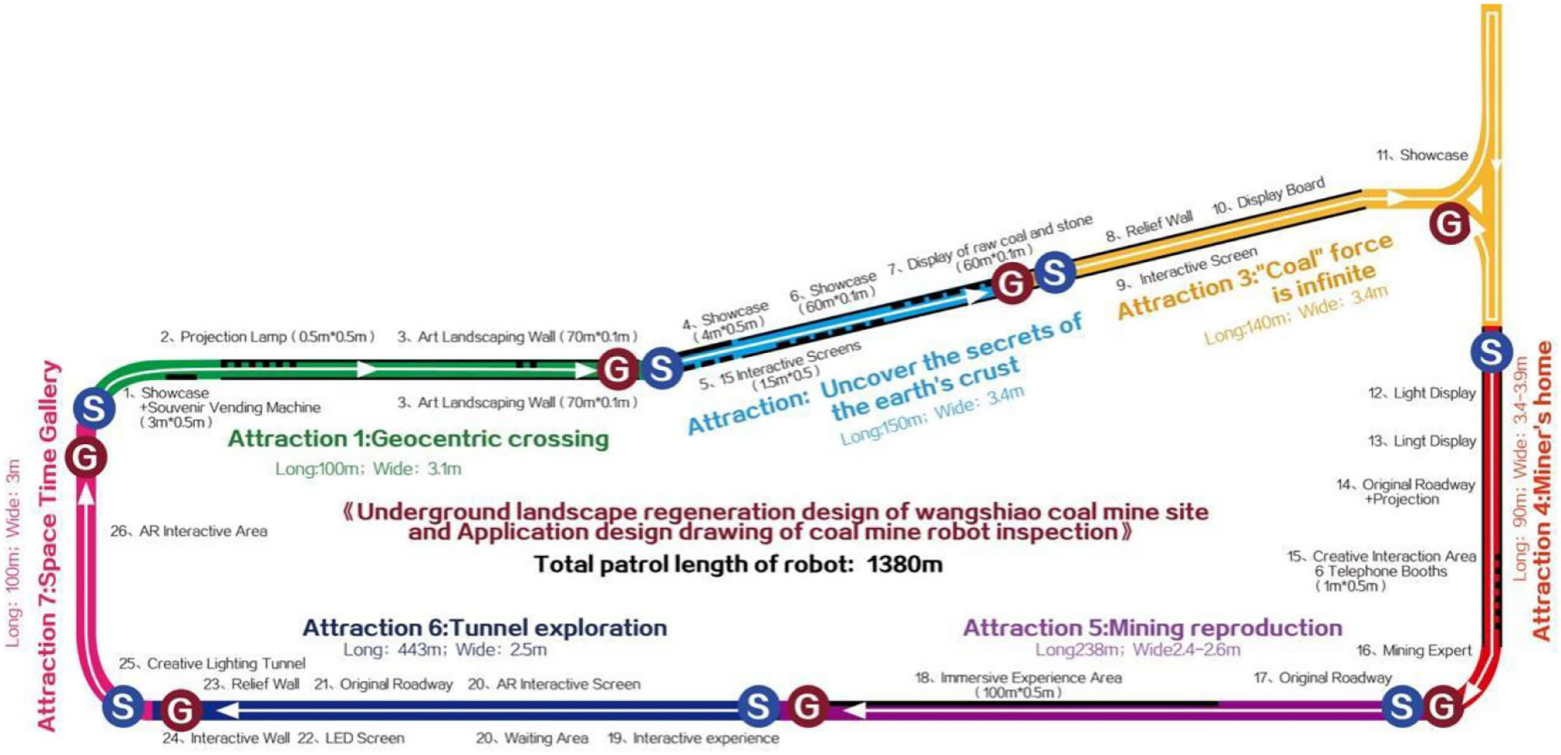
Motion trajectory of inspection robot.

The underground tour line of industrial heritage of Wangshiwa coal mine is 1380 m long, with an average roadway width of 3.2 m (as shown in Figure 11), and can accommodate 200 visitors for 2 h. The landscape design of underground roadway includes 7 plates: Geocentric crossing (total length: 100 m, width: 3.1 m), Crustal disclosure (total length: 150 m, width: 3.4 m), Unlimited “coal” force (total length: 140 m, width: 3.4 m), Miner's home (total length: 90 m, width: 3.4–3.9 m), Mining reproduction (total length: 238 m, width: 2.4–2.6 m), Tunnel exploration (total length: 443 m, width: 2.5 m), Space time gallery (100 m long and 3 M wide), as shown in Figure 12. An intelligent inspection robot is placed in each landscape node area. An optimal inspection line is set according to the landscape length and width of each roadway.

According to the underground planning of the industrial site, the inspection robot is mainly applied to the substation, east lane, sedimentation tank, and excavation face and disaster avoidance chamber. The layout is shown in Figure 11.

The speed of intelligent inspection robot is 3.6–4.5 km/h. Through practical calculation and application, it is found that the average time of inspection in each landscape node is 3–5 min, which greatly reduces the labor cost and improves the operation efficiency and personnel safety. The path and line planning are shown in Figure 12.

Coal mine wasteland is a special landscape type after a large number of mineral resources are exhausted (Xin and Zeng, 2021), and post industrial areas are often regarded as potential leisure areas (Loures et al., 2011; Rostański, 2021). China is at a critical stage where the contradiction between mine economic development and mine environment deterioration is prominent (Guan et al., 2017). In the protection and reuse of coal mine industrial heritage, we should follow its authenticity principle (Chylinska and Kołodziejczyk, 2017), and maximize its material and intangible cultural heritage through intelligent means (Bai, 2020). The unique landscape of coal industry heritage will be better protected and utilized through intelligent technology.

Intelligent inspection is an important part of intelligent protection of industrial heritage. Based on 5G technology (Zhen et al., 2021), intelligent inspection robot is introduced to replace manual inspection, which can improve inspection frequency and quality, ultimately ensure stable and efficient operation of equipment, and reduce cost and increase efficiency. At the same time, it can improve the overall level of heritage protection, so that the management of industrial heritage protection is more scientific, intelligent and refined.

## Advantages and Disadvantages of Inspection Robot

Regarding the path planning, motion obstacle avoidance, and inspection detection of the inspection robot, A^*^ algorithm, artificial potential field method, sensor detection are used to realize the corresponding functions, respectively. The advantages and disadvantages of these methods are as follows.

(1)A^*^ **algorithm**. A^*^ algorithm gradually determines the next path grid by comparing the heuristic function values of the neighbors of the current path grid. When the number of searched points is large and the search range is large, the optimal solution of the path can be obtained, but the efficiency is lower. When there are multiple minimum values, A^*^ algorithm cannot ensure the optimal path.

**(2) Artificial potential field method**. The artificial potential field method is to construct an artificial potential field to simulate the action mechanism when the starting point, end point and obstacle position are known. The advantage of this method is that it is actually a feedback control strategy, which has certain robustness to control and sensing errors. The disadvantage is that there is a local minimum problem, so it is not guaranteed to find the solution of the problem.

The main problems are as follows.

(a) When the object is far away from the target point, then the virtual attractive force will become particularly large, and the relatively small repulsive force even can be ignored. Then the robot may encounter obstacles on the motion path.

(b) When there is an obstacle near the target point, the repulsive force will be very large and the attractive force is relatively small, and it is difficult for the robot to reach the target point.

(c) At some points, the attractive force and the repulsive force are exactly equal in magnitude and opposite in direction. Hence, it is prone to fall into a local optimal solution or oscillation.

**(3) Sensing detection**. According to the needs of the site, through the use of various sensors, various indicators can be detected to realize early warning, and the corresponding signals can be transmitted to the operation and maintenance management cloud platform to manage the robot inspection tasks, so as to realize the real-time state control of the robot. However, the price of sensor equipment is generally high, and the communication problem between sensor and upper management platform needs to be solved by professionals.

**(4) Advantages and disadvantages of inspection robot**

**Advantages**. Aiming at the transformation and protection of coal mine industrial heritage, the intelligent robot is the core to realize the inspection task. So the intelligent inspection and management can be promoted faster. It is conducive to reducing labor costs, improving staff work efficiency, and ensuring safe and stable operation.

**Disadvantages**. In the inspection process of the robot, the power consumption of the robot is fast. When performing multiple inspection tasks, the robot may have insufficient power. If the robot realizes automatic charging, it is required to independently detect the power and navigate to the charging pile autonomously to realize autonomous charging, which adds some new requirements and increases the use cost.

In addition, the intelligent inspection robot has high requirements for hardware and software system configuration. It needs more material and technical support.

## Conclusion

This paper briefly describes the development process of Wangshiwa coal mine in Tongchuan City, Shaanxi Province, China. The coal mine has rich historical, social, artistic, technological and spiritual industrial heritage values. In this regard, the industrial heritage is reformed and protected, the key protection scope is analyzed, and the corresponding protection strategy is put forward to realize the safe and efficient utilization of coal mine heritage. Aiming at the problems such as long inspection interval, difficulty in troubleshooting and untimely warning caused by traditional manual inspection of key facilities and equipment in underground, the intelligent inspection robot system is proposed to be applied in underground inspection. This paper describes the system structure, operation principle, and function of each intelligent inspection robot subsystem, as well as its construction path and method. The research shows that the intelligent inspection robot can realize scientific, intelligent and fine management of industrial heritage protection, which provides guidance for intelligent protection of coal mine industrial heritage.

## Data Availability Statement

The original contributions presented in the study are included in the article/supplementary material, further inquiries can be directed to the corresponding author.

## Author Contributions

YS was responsible for manuscript writing and analysis. YL was responsible for manuscript review and editing. ZL proposed the idea and was responsible for the literature search. All authors contributed to the article and approved the submitted version.

## Funding

This research was funded by the Scientific Research Plan Projects of Education Department of Shanxi Province (No. 18JK0725) and the Social Development Grant of Shanxi Province (No. 2019k020).

## Conflict of Interest

ZL was employed by Xi'an Edson Landscape Design Co., Ltd. The remaining authors declare that the research was conducted in the absence of any commercial or financial relationships that could be construed as a potential conflict of interest.

## Publisher's Note

All claims expressed in this article are solely those of the authors and do not necessarily represent those of their affiliated organizations, or those of the publisher, the editors and the reviewers. Any product that may be evaluated in this article, or claim that may be made by its manufacturer, is not guaranteed or endorsed by the publisher.
